# The Components of Interpersonal Synchrony in the Typical Population and in Autism: A Conceptual Analysis

**DOI:** 10.3389/fpsyg.2022.897015

**Published:** 2022-06-06

**Authors:** Claire Bowsher-Murray, Sarah Gerson, Elisabeth von dem Hagen, Catherine R. G. Jones

**Affiliations:** ^1^Wales Autism Research Centre, School of Psychology, Cardiff University, Cardiff, United Kingdom; ^2^Cardiff University Centre for Human Developmental Science, School of Psychology, Cardiff University, Cardiff, United Kingdom; ^3^Cardiff University Brain Imaging Research Centre, School of Psychology, Cardiff University, Cardiff, United Kingdom

**Keywords:** interpersonal synchrony, behavioural co-ordination, social interaction, synchronisation, social motor behaviour, autism

## Abstract

Interpersonal synchrony – the tendency for social partners to temporally co-ordinate their behaviour when interacting – is a ubiquitous feature of social interactions. Synchronous interactions play a key role in development, and promote social bonding and a range of pro-social behavioural outcomes across the lifespan. The process of achieving and maintaining interpersonal synchrony is highly complex, with inputs required from across perceptual, temporal, motor, and socio-cognitive domains. In this conceptual analysis, we synthesise evidence from across these domains to establish the key components underpinning successful non-verbal interpersonal synchrony, how such processes interact, and factors that may moderate their operation. We also consider emerging evidence that interpersonal synchrony is reduced in autistic populations. We use our account of the components contributing to interpersonal synchrony in the typical population to identify potential points of divergence in interpersonal synchrony in autism. The relationship between interpersonal synchrony and broader aspects of social communication in autism are also considered, together with implications for future research.

## Introduction

The tendency for social partners to temporally co-ordinate their behaviour, known as interpersonal synchrony (IS), is a common feature of social interactions (Bernieri et al., 1988; Delaherche et al., 2012). It is sometimes the product of conscious effort, such as when we shake hands, high five, or dance together. It may also arise spontaneously: social partners might fall into step (Zivotofsky and Hausdorff, 2007), align their postural positions (Shockley et al., 2003; Gaziv et al., 2017), or entrain their body movements (Hadar et al., 1984) or facial expressions (Louwerse et al., 2012). IS may display rhythmical properties (e.g., walking in step; nodding), but equally may be less structured in nature (e.g., sporadic gestures or postural adjustment). Although IS may arise *via* a broad range of behavioural processes including gesture, gaze, facial expression, speech, and vocalisation, the current review is focussed on the synchrony of non-verbal behaviours. Temporal co-ordination of social behaviour emerges shortly after birth (Condon and Sander, 1974; Dominguez et al., 2016) and becomes more temporally accurate, more complex, and less reliant on adult facilitation during infancy (Hilbrink et al., 2015; Meyer and Hunnius, 2020). Mother-child IS is believed to positively influence self-regulation and empathy, and to promote later cognitive, social and emotional development and secure attachment relationships (Harrist and Waugh, 2002; Feldman, 2007; Evans and Porter, 2009). Throughout the lifespan, IS serves as a social signifier and promotes various social outcomes, including increased affiliation (Hove and Risen, 2009; Tunçgenç et al., 2015), rapport (Vacharkulksemsuk and Fredrickson, 2011), bonding (Tarr et al., 2015; Tunçgenç and Cohen, 2016), helping (Tunçgenç and Cohen, 2018), and co-operation (Rabinowitch and Meltzoff, 2017). Such effects are present both when IS is spontaneous and when it is intentional, although there is mixed evidence as to whether they are enhanced when partners share an intention to co-ordinate (Reddish et al., 2013; Howard et al., 2021). The full range of social outcomes arising from IS has been documented in recent reviews (Rennung and Goritz, 2016; Vicaria and Dickens, 2016; Mogan et al., 2017; Cross et al., 2019; Hoehl et al., 2021).

Difficulties with social communication and social interaction, including with non-verbal communicative behaviour and building and maintaining relationships, are hallmarks of autism (American Psychiatric Association, 2013). As such, there has been particular interest in how autistic people engage in and experience IS (McNaughton and Redcay, 2020). Evidence indicates that IS is less accurate and/or less frequent in interactions involving autistic people, in both spontaneous (Marsh et al., 2013; Fitzpatrick et al., 2016; Kaur et al., 2018; Georgescu et al., 2020; Zampella et al., 2020) and intentional (Fitzpatrick et al., 2016) contexts. Several studies have found an association between lower levels of IS and higher levels of autistic traits (Brezis et al., 2017; Cheng et al., 2017; Fitzpatrick et al., 2017b; Zampella et al., 2020; Granner-Shuman et al., 2021; although cf. Kaur et al., 2018). There is also some evidence that the social significance of IS may be attenuated for many autistic people (Koehne et al., 2016).

IS emerges as a function of multiple mechanisms operating in concert with each other (Konvalinka et al., 2010; Delaherche et al., 2012; Mills et al., 2019; McNaughton and Redcay, 2020). However, research commonly focuses on the role of individual mechanisms, such as attention (e.g., Temprado and Laurent, 2004; Richardson et al., 2007), perceptual processing (e.g., Noel et al., 2018), motor behaviour (e.g., Hart et al., 2014; Monier and Droit-Volet, 2019) and social factors (e.g., Kirschner and Tomasello, 2009; Lumsden et al., 2012; Honisch et al., 2021). To better understand IS there is a need to synthesise findings from across the perceptual, sensorimotor, social and cognitive domains. Understanding how the component processes underlying IS operate together is also necessary for understanding why IS manifests differently in autism. In addition to core differences in social functioning, differences between autistic and typical populations have been observed across domains relevant to IS, including attention (Frazier et al., 2017; Hedger et al., 2020), temporal perception (e.g., Allman and Falter, 2015); perceptual processing (e.g., Feldman et al., 2018; Meilleur et al., 2020), and motor behaviour (e.g., Fournier et al., 2010). Therefore, characterising IS in autism necessarily requires a holistic understanding of how differences in functioning across multiple underlying processes operate together.

**FIGURE 1:**
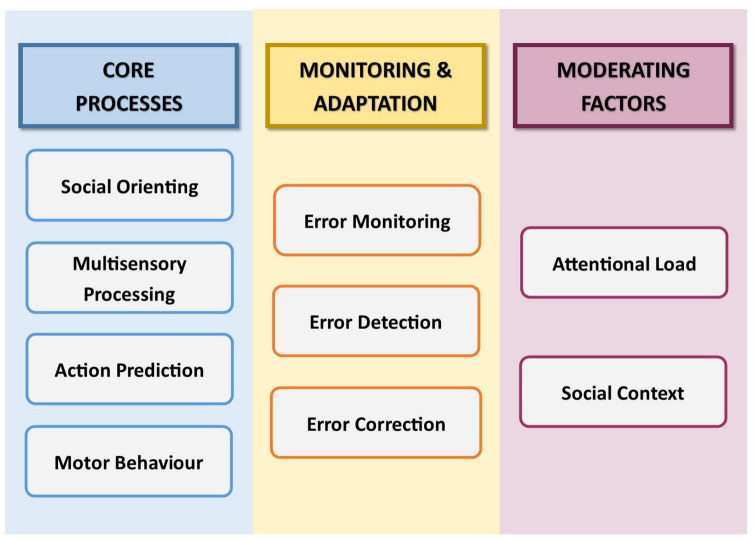
Schematic illustration of the components of non-verbal interpersonal synchrony, as discussed in this review.

The first part of this conceptual analysis begins with a synthesis of the key component mechanisms that contribute to IS (Figure 1), including an exploration of how such processes interact, and the factors that may moderate their operation. Where useful, we additionally draw on the related concepts of imitation and joint action. Imitation, like IS, involves behavioural matching in form, albeit not in time (Hove and Risen, 2009; Catmur and Heyes, 2013). Joint action involves the conscious co-ordination of complimentary behaviour to achieve a shared goal (Sebanz et al., 2006). The parallels between these two phenomena and IS mean that their underlying processes can shed light on the role of equivalent processes in IS. The second part of this conceptual analysis describes how each of the identified component mechanisms operates in autism, and considers the extent to which differences in the functioning of these mechanisms may explain differences in IS. In considering each mechanism not only individually but also as part of a wider system, we aim to build an understanding of how relevant mechanisms collectively underpin reduced IS in autism.

## Part 1: Interpersonal Synchrony in the Typical Population

### Social Orienting

In order for an individual to synchronise their movements with an external stimulus, they must have perceptual access to the stimulus (Richardson et al., 2007; Oullier et al., 2008; Miyata et al., 2017; Oh Kruzic et al., 2020). Perception of the stimulus, in turn, requires that sufficient attentional resources are allocated to it (Aubin et al., 2021). In the case of IS, the relevant stimulus is one’s interaction partner. During social interaction, the perception of one’s partner and their movements is facilitated by an inherent tendency to orient toward social stimuli, that is, the inherent preferential allocation of visual attention to social rather than non-social cues (Fletcher-Watson et al., 2008; Gluckman and Johnson, 2013; Leppanen, 2016; Rösler et al., 2017). Experimental manipulation of the extent to which partners are oriented toward each other has demonstrated its importance in facilitating IS. Partners spontaneously synchronised their movements at above chance levels when looking directly at each other, but not when they had only peripheral visual access to each other (Richardson et al., 2007).

It is also important to consider what aspects of a social partner’s behaviour are particularly informative. The direction of a partner’s gaze and the kinematic cues provided by their limb movements convey information about their future movements (Sartori et al., 2011; Ansuini et al., 2014; Khoramshahi et al., 2016), particularly where the end of point of an action is otherwise unclear (Fulceri et al., 2018). Such cues are likely to facilitate both spontaneous and intentional IS. Mutual gaze between partners may also support the emergence of IS. Although there is no direct evidence that mutual gaze plays a role in IS, mutual gaze is considered to facilitate social engagement, and is known to promote spontaneous partner imitation (Wang and Hamilton, 2012; Prinsen et al., 2017). The shared element of behavioural matching in IS and in imitation suggests that mutual gaze may have a similar role in promoting IS.

Flexibility in attending to social cues is another key consideration for effective IS. Naturalistic interactions almost always contain multiple social cues, occurring either simultaneously or in quick succession. To capitalise on their predictive value, it seems likely that attention must be flexibly directed and redirected toward relevant cues. A further consideration is that social cues often consist of limb or whole-body movements, which are inherently dynamic. For example, in order for a handshake to be successfully synchronised, each partner must match their eye movements to the movement of the other’s hand, such that the motion of the other partner is efficiently tracked *via* smooth pursuit (Johnson et al., 2016). In summary, social orienting is a core process necessary for IS to take place. Dynamically attending to a partner’s behavioural cues, particularly kinetic and eye gaze cues, is likely to be the specific means by which attending to a social partner supports IS.

### Multisensory Processing

Interacting with a social partner is a multisensory experience, involving the integration of social information delivered *via* multiple modalities. Sensory inputs from two or more modalities that occur with sufficient temporal proximity are perceptually combined *via* multisensory integration (Alais et al., 2010; Murray et al., 2016). There is evidence that efficient multisensory integration supports IS. For example, synchronisation of both fine and gross motor activity was achieved with significantly greater accuracy when multisensory stimuli were available, as compared with when stimuli were unisensory only (Elliott et al., 2010; Su, 2014; Wright et al., 2014; Khan et al., 2020). Further, increased multisensory acuity was significantly associated with increased spontaneous IS (Noel et al., 2018). Multisensory integration of social information is likely to support IS in a number of ways. First, it likely enhances the perceptual cohesion and salience of the behaviour (Bahrick and Todd, 2012) with which synchrony is to be achieved. Second, the combination of information from multiple streams enables the individual to make enhanced statistical predictions about the stimulus (Elliott et al., 2010). Third, multisensory integration may promote perception action coupling, whereby audio-visual cues are likely to engage the observer’s own sensorimotor system more than single modality cues, which then simulates the observed movement as a means of predicting its ongoing trajectory (Su, 2014). Fourth, in addition to supporting the processing of incoming social stimuli, multisensory integration is likely to support the effective planning, monitoring and adjustment of one’s *own* actions, which requires the effective integration of visual and proprioceptive information about one’s own motor behaviour (Foster, 2019). Overall, therefore, efficient multisensory processing is a core process that likely supports effective IS *via* multiple pathways.

### Action Prediction

A defining feature of IS is that partners move together simultaneously. In order to achieve this, a partner’s movements must be anticipated, both spatially and temporally (Sebanz and Knoblich, 2009; Meyer and Hunnius, 2020; Gvirts Probolovski and Dahan, 2021), rather than merely reacted to. The tendency to make spontaneous, online predictions about the actions of others is present throughout the lifespan (Flanagan and Johansson, 2003; Reddy et al., 2013; Sebanz and Knoblich, 2021), and is likely influenced by low level kinematic information (Ansuini et al., 2014) and gaze cues (Khoramshahi et al., 2016), as well as higher-level cognitive processes, such as assessing a partner’s likely intentions in light of other contextual information (Falck-Ytter, 2012). A number of studies suggest a role for action prediction in intentional IS. For example, participants who displayed more accurate temporal prediction in a solo task showed significantly greater accuracy and stability of synchronisation during a joint finger tapping task (Pecenka and Keller, 2011). Similarly, toddlers who made more temporally accurate predictions in an observation task moved with greater temporal stability during a joint action task with a partner (Meyer et al., 2015). Thus, the evidence suggests that being able to form accurate predictions about a partner’s movements is a core process facilitating intentional IS. There is no direct evidence as to how action prediction abilities inform spontaneous IS. However, some form of prediction must necessarily occur in order for behaviour to become temporally aligned (Gvirts Probolovski and Dahan, 2021). Further research is required to establish exactly how predictions are made and integrated with other component processes in the context of spontaneous IS.

### Motor Behaviour

In addition to anticipating the movement of a partner, it is necessary to plan and execute one’s own complimentary movement sequence, both temporally and spatially. The ability to synchronise simple motor movements with an external stimulus is limited in young children, partly by a relatively limited capacity to adapt motor behaviour to the tempo of an external stimulus, but develops during childhood (Drake et al., 2000; McAuley et al., 2006; Monier and Droit-Volet, 2018) as a function of developing motor skills (Monier and Droit-Volet, 2019). Immature motor skills are therefore believed to limit young children’s levels of IS (Trainor and Cirelli, 2015), although there is limited direct evidence as to the role of motor skills in IS in typical populations. In support of a role for motor planning in intentional IS, reduced performance on a motor planning task was significantly associated with reduced intentional IS in a hand movement task (Granner-Shuman et al., 2021). However, the contribution to IS of other aspects of motor abilities are yet unknown.

Just as motor skills are likely important to the achievement of IS, so too is the form of movement people tend to produce. People tend to display an “individual motor signature,” which is a distinct and stable pattern of movement that is personal to the individual in terms of direction, range and velocity of movement (Richardson and Johnston, 2005; Hart et al., 2014; Słowiński et al., 2016). Some individual motor signatures convey more predictive information than others (Koul et al., 2016), which is likely to make them easier to synchronise with. Further, evidence suggests that partners with similar individual motor signatures are better at predicting the timing of each other’s movements (Colling et al., 2014) and achieve a higher degree of co-ordination when moving together (Słowiński et al., 2016), relative to partners whose motor signatures are relatively dissimilar. As well as displaying individualised patterns of movement, people also tend to exhibit a preferred *pace* of movement, or spontaneous motor tempo (Delevoye-Turrell et al., 2014). Just as the (dis)similarity of partners’ motor signatures influences the degree of co-ordination they achieve when interacting, it seems likely that those with relatively similar spontaneous motor tempos would achieve higher levels of IS than those with relatively dissimilar motor tempos. In sum, interacting partners’ motor abilities, their natural movement patterns as well as, potentially, their relative pace of movement, all contribute to IS.

### Monitoring and Adaptation

The component processes of IS have so far been considered independently. However, during dynamic real-world interactions involving both intentional and spontaneous IS, these processes are believed to be embedded together in a continuous feedback loop (Shamay-Tsoory et al., 2019; Gvirts Probolovski and Dahan, 2021). Specifically, it is proposed that predictions about a partner’s movement and one’s own plan to align with it are integrated into a forward model of the shared movement between partners. As motor commands are executed, “error monitoring” occurs, whereby both one’s own and one’s partner’s actual motor output is compared to the forward model. “Error detection” occurs when either partner’s actual movement does not match the generated prediction. Error detection precipitates “error correction,” where the predictive model and movement plan are updated (Shamay-Tsoory et al., 2019).

The component processes of IS, described above, may contribute to the effectiveness of the feedback loop in a number of ways. For example, effective error monitoring is likely underpinned by continued social orienting and dynamic attendance to behavioural cues. Further, error monitoring, in the context of IS, consists of detecting asynchrony between the actions of partners. Thus, perceptual sensitivity to the temporal alignment of events likely contributes to the achievement of IS. Error correction is likely to draw on action prediction and motor abilities. The effectiveness of the updating process also depends on how quickly it occurs (Vishne et al., 2021). The faster the internal model and movement plan can be updated and implemented, the more closely aligned partners’ behaviour will be over time. Efficient updating is likely to be critical in real-world social interactions, in which the form and speed of partners’ movements change over time, placing persistent demands on adaptive mechanisms.

The process of continuous mutual adaptation during IS, described by the above account, is supported by behavioural evidence. For example, dyads required to synchronise their finger tapping adjusted the time between their taps in opposite directions to one another, on a tap-by-tap basis (Konvalinka et al., 2010), suggesting that each partner continuously accounted for the pace of the other and modified the pace of their own tapping accordingly. During more complex interactions, there is evidence that partners spontaneously adapt both the spatial (Sartori et al., 2009; Candidi et al., 2015) and temporal (Vesper et al., 2011) qualities of their movements, so as to make them more predictable to their partner. The result of partners’ mutually adaptive behaviour is that they coalesce into a third movement pattern distinct from either of their individual motor signatures (Hart et al., 2014). Overall, this line of research emphasises that the perceptual and motor abilities of each partner operate within a dynamic context of bidirectional adjustment and adaptation.

### Attentional Load

During a social interaction, attentional resources are subject to demands from multiple sources. For example, while partners process visual information about their partner they will also be processing the content of their conversation and making inferences about the other person’s mental state (Westra and Nagel, 2021). There may also be input from distractors in the environment (e.g., an interesting visual display or an overheard conversation), sensory input (e.g., feeling too hot or too cold), or other unrelated thoughts. The distribution of attention across multiple stimuli can influence the extent of intentional IS. Participants asked to synchronise arm movements were more accurate in their synchrony when attending to the task, compared to attending to a simultaneous reaction time task, or sharing attention across tasks (Temprado and Laurent, 2004). The extent to which distractors are present during real life interactions might similarly moderate levels of IS. However, it is notable that participants in this research were explicitly instructed to direct their attention away from IS-relevant stimuli. In real-world interactions, social cues are preferentially attended to (see the section “Social Orienting”) and processed preferentially even when not task-relevant (Lavie et al., 2003). Preferential processing of social stimuli may mean that intentional IS is relatively unaffected when distractors are present, or affected only when distractors are particularly salient.

From a different perspective, it has been proposed that spontaneous IS might itself arise as a means of minimising overall attentional load (Koban et al., 2019). When IS arises (either spontaneously or intentionally), a social partner’s actions are relatively similar in time and form to one’s own, such that they are easier to predict and require less effortful processing. This, in turn, is likely to mean that greater attentional resources are available for processing other stimuli. However, given that intentional IS is an effortful process whereas spontaneous IS is not, spontaneous and intentional IS may relate differently to attentional load (Aubin et al., 2021). Further research is required to determine how attentional load and IS influence each other each other during everyday interactions.

### Social Context

A number of studies have considered the role of social context in synchronisation, with some finding evidence of greater synchronisation when participants synchronise with a social stimulus (such as another person) as compared with a non-social stimulus (such as a mechanical arm) (Kirschner and Tomasello, 2009; Honisch et al., 2021; Howard et al., 2021). One interpretation is that the very existence of a social context motivates individuals to synchronise (Kirschner and Tomasello, 2009; Yu and Myowa, 2021). An alternative interpretation is that the perceived engagement of a partner, rather than their mere presence, provides increased motivation to synchronise. This explanation is supported by evidence that participants synchronised more accurately with a social partner than with a non-social stimulus, even when a social partner was present in both conditions (Howard et al., 2021). Another possibility, however, is that social stimuli provide greater congruency with the action to be produced by the participant, relative to non-social stimuli (Honisch et al., 2021; Howard et al., 2021). Studies that employed identical social and non-social stimuli (e.g., computer based, auditory signals), apart from being described to participants as originating either from a human partner or a computer, found that rates of synchronisation were comparable between conditions (Koehne et al., 2016; Mills et al., 2019). This suggests that the fact that participants thought they were interacting with a social partner, as opposed to a non-social stimulus, provided *no* intrinsic motivational effect. However, where a partner’s actions are represented only by computer-based signals, the “presence” of the social partner is much less salient than in a more naturalistic interaction. This limited salience is a possible alternative explanation for lack of difference between conditions. Thus, the relative contributions of social context and physical congruency on IS remain unclear.

The studies described above considered whether social presence motivated synchronisation at the group level. An alternative approach is to examine how individual differences in trait levels of social motivation influence levels of IS. Using a self-reported measure of social motivation, participants classified as “pro-social” were found to spontaneously synchronise with a partner to a significantly greater extent than participants classified as “pro-self” (Lumsden et al., 2012), suggesting that higher social motivation at an individual level precipitates higher levels of IS.

The quality of the social relationships between partners may also affect IS. Partners with pre-existing affiliative relationships have been found to synchronise more than unfamiliar partners (Latif et al., 2014). Further, whether participants are positively or negatively disposed toward previously unfamiliar partners influences levels of IS. For example, participants spontaneously synchronised significantly more with partners they believed to be punctual (Miles et al., 2010), honest (Brambilla et al., 2016), and attractive (Zhao et al., 2015), relative to partners they believed to be tardy, dishonest, and unattractive, respectively. Collectively, the effects of social moderators on IS has led researchers to conclude that IS is influenced by the need or desire to make social connections with others (Lumsden et al., 2014; Hoehl et al., 2021) and serves as a means of co-constructing a social space (Cornejo et al., 2017).

Although there is relatively strong evidence that social context modulates IS, less is known about the mechanism by which it does so. One possibility is that the motivation to seek connection with a partner influences social orienting, such that the increased desire to connect with a partner increases attentional allocation to them (Lumsden et al., 2012; Gvirts and Perlmutter, 2020). Increased attention has been proposed to have cascading effects on other component processes, such as improved action prediction, leading to more accurate motor planning. Critically, the resulting behavioural alignment is thought to be experienced as rewarding, thus promoting continued mutual social attention and maintaining the integrity of the feedback loop described above (Kokal et al., 2011; Shamay-Tsoory et al., 2019; Gvirts and Perlmutter, 2020). Feelings of reward may arise because behavioural alignment leads to reduced processing demands (Koban et al., 2019; Shamay-Tsoory et al., 2019) but are also likely to be influenced by the social significance of the interaction to each partner. The extent to which IS is experienced as rewarding by each partner is therefore a likely further source of variation in levels of IS (Gvirts and Perlmutter, 2020; Gvirts Probolovski and Dahan, 2021).

## Part 2 – Interpersonal Synchrony in Autism

Converging evidence indicates that, on average, IS is reduced in autism (McNaughton and Redcay, 2020). Studies employing structured experimental tasks involving pendulum swinging (Fitzpatrick et al., 2016), chair rocking (Marsh et al., 2013), movement improvisation (Brezis et al., 2017) and gaze following (Liu et al., 2021) all found lower levels of IS when one of the interacting partners was autistic, relative to when both partners were non-autistic. A similar pattern of results has emerged from the analysis of naturalistic interactions. IS was reduced during a clinical diagnostic interview for adults who were subsequently given an autism diagnosis, compared to those who were not (Koehler et al., 2021). Similarly, conversations between dyads in which at least one partner was autistic were characterised by reduced IS, relative to conversations between typical dyads (Georgescu et al., 2020). In typical adults, higher levels of autistic traits within dyads were significantly associated with reduced spontaneous motor synchrony when partners walked and talked together (Cheng et al., 2017). Thus, there is a range of evidence suggesting reduced IS in autism. However, a substantial majority of studies that support this conclusion compared IS in mixed dyads (consisting of one autistic and one non-autistic partner) with IS in typical dyads. Relatively little is known about levels of IS in interactions between autistic people.

Additionally, a feature of many of the studies described above is that they involved a relatively sophisticated level of social interaction. By contrast, autistic and non-autistic participants achieved comparable levels of synchrony in an interaction in which the social, perceptual, and motoric content of the interaction was substantially reduced, in that it involved only the exchange of signals with an unseen partner *via* a computer button press (Koehne et al., 2016). Together with evidence that synchrony is reduced but still present at above chance levels in more complex interactions (Georgescu et al., 2020; Koehler et al., 2021), this finding suggests that a basic tendency to synchronise may be intact in autism. However, it is unclear which particular processes may account for the differences in IS during naturalistic social interactions. This section explores the potential points of divergence in IS between autistic and non-autistic individuals.

### Social Orienting

Atypical social orienting has been proposed as one possible mechanism precipitating reduced IS in autism (Fitzpatrick et al., 2016; Brezis et al., 2017; McNaughton and Redcay, 2020). Recent meta-analyses have found that, on average, autistic individuals display reduced visual attention toward social stimuli relative to non-autistic individuals (Frazier et al., 2017; Hedger et al., 2020). However, there is also heterogeneity between studies, with a substantial number finding no differences in the tendency to visually attend to social stimuli (Frazier et al., 2017; Hedger et al., 2020). Further, a majority of studies examining social orienting involve passive viewing of stimuli, rather than social orienting during live interactions, which may prompt different patterns of gaze behaviour (von dem Hagen and Bright, 2017). There is less evidence of social orienting in autism during active engagement in an interaction. While some research reports an increased tendency amongst autistic individuals to visually attend to background information rather than to a social partner (Zhao et al., 2021), others have failed to observe such an effect (Canigueral and Hamilton, 2019). There is more substantial evidence that autistic people attend atypically to specific cues, with evidence, for example that mutual gaze (Jones and Klin, 2013; Vabalas and Freeth, 2016; Nyström et al., 2017; Hessels et al., 2018; McParland et al., 2021) and gaze following (Vivanti et al., 2011; Riby et al., 2013) are, on average, reduced in autism.

There is also evidence of heterogeneity within the *patterns* of social attending displayed by autistic people. For instance, while non-autistic participants viewing video footage of social interactions had highly predictable looking patterns, the gaze patterns of autistic participants were highly variable, and became less similar to typical gaze patterns with higher levels of autistic traits (Avni et al., 2020). Additionally, differences in social orienting may be moderated by gender, with evidence that autistic females and neurotypical individuals display comparable patterns of visual attention toward faces (Harrop et al., 2018; Harrop et al., 2019). However, autistic females are more likely than autistic males to employ strategies to display neurotypical social behaviour and/or compensate for social difficulties (Cook et al., 2021b). Thus, the moderating effect of gender in these studies may have resulted from female participants employing a learned strategy through which they consciously attend to faces (Harrop et al., 2019). Recent studies have also considered how social orienting proceeds over time during the course of an interaction. Autistic and non-autistic participants both displayed a high probability of initial visual attending to social stimuli, followed by a decline after several seconds. However, non-autistic participants were significantly more likely than autistic participants to return their visual attention to the social stimuli shortly afterward (Del Bianco et al., 2021; Hedger and Chakrabarti, 2021). Further, autistic children shifted their gaze in response to the gaze of a social partner significantly more slowly that non-autistic children (Liu et al., 2021). Longer latencies in gaze following are likely to reduce the extent to which relevant behavioural cues can be perceived and acted upon.

Overall, the evidence indicates that that some, but not all, autistic individuals are likely to demonstrate atypical social orienting, with patterns of visual attention to social stimuli unfolding differently over time. Given that social orienting facilitates IS in typical populations, it is likely that differences in social orienting over the course of an interaction play a role in reduced IS for some autistic individuals. Although there is currently no direct evidence to this effect, research in the related field of imitation provides some indicative support. For instance, reduced visual attention to a demonstrator by autistic children was significantly associated with reduced spontaneous imitation of the acts performed by the demonstrator (Gonsiorowski et al., 2015). Further, when explicitly instructed to pay attention to the features of an action, autistic and non-autistic participants imitated the action with an equivalent degree of accuracy (Gowen et al., 2020). Thus, atypical visual attention to a partner influences imitation in autism, and is likely to play an equivalent role in the context of IS.

### Multisensory Processing

As discussed in Part 1, efficient multisensory integration of social stimuli is likely to support the emergence of IS. The balance of evidence suggests that autistic individuals demonstrate reduced multisensory acuity, reflected in an increased tendency to report relatively asynchronous visual and auditory stimuli as originating from the same source (Zhou et al., 2018; Wallace et al., 2020). This can potentially lead to inappropriate perceptual binding of incoming sensory stimuli and thus a less coherent picture of the immediate environment (Casassus et al., 2019), including IS-relevant social cues. However, a substantial minority of studies observe no difference in multisensory processing abilities between autistic and non-autistic participants (Feldman et al., 2018; Meilleur et al., 2020; Wallace et al., 2020). Possible explanations for this divergence include variation in the age of participants, with some researchers suggesting that maturation of multisensory integration is delayed in autism (Beker et al., 2018; Feldman et al., 2018). Additionally, there is more consistent evidence of differential multisensory processing when complex, speech-based stimuli are used, relative to simplified, non-social stimuli such as flashes and tones (Stevenson et al., 2014; Meilleur et al., 2020).

To our knowledge, only one study to date has examined the relation between multisensory processing and IS in autism, with autistic children displaying both reduced audio-visual multisensory acuity and reduced non-verbal synchrony, relative to typically developing children (Noel et al., 2018). However, the multisensory acuity of autistic children was not significantly associated with the amount of IS they displayed, potentially indicating that autistic participants did not make use of available audio-visual information to inform other component processes of IS (Noel et al., 2018).

As noted in relation to typical populations above, multisensory integration of proprioceptive and visual information may also be important in supporting IS, because it enables the effective monitoring of one’s own motor behaviour. There is evidence that overreliance on proprioceptive information leads to less efficient multisensory integration of proprioceptive and visual information in autism (Greenfield et al., 2015), and that this precipitates reduced accuracy in motor behaviour (Glazebrook et al., 2009; Haswell et al., 2009). However, there is no evidence, to date, of an association with reduced IS specifically.

Overall, there is evidence of atypical multisensory processing in autism, which is likely to contribute to reduced IS. However, further research is required to establish the extent of its contribution and its relationship with other component processes.

### Action Prediction

Evidence from typical populations, as outlined in Part 1, indicates that IS is facilitated by accurately anticipating the spatial and temporal aspects of a social partner’s movement. In autism, several researchers have proposed that a generalised impairment in prediction underpins a variety of autistic traits (Sinha et al., 2014; Van de Cruys et al., 2014; Cannon et al., 2021), including reduced action co-ordination (e.g., Cerullo et al., 2021). There is evidence of atypical action prediction in autism, which potentially influences IS. For example, when observing the repeated actions of a cartoon character, autistic children generated fewer and less accurate spontaneous action predictions than non-autistic children (Schuwerk et al., 2016). In more naturalistic contexts, autistic individuals have displayed a reduced tendency to make spontaneous action predictions about others’ behaviour from both gaze (Pierno et al., 2006) and kinematic (Hudson et al., 2021) cues. Accuracy of action prediction may depend not only on the ability of the person making the prediction, but also on who is being observed (Cook, 2016). There is evidence that, when predicting the actions of another based on observing their movement kinematics, autistic people are better at predicting the actions of other autistic people than they are at predicting the actions of non-autistic people, and vice versa (Montobbio et al., 2022). This suggests that, when autistic and non-autistic partners interact, they may experience bidirectional difficulties with action prediction, potentially leading to difficulties in establishing and maintaining IS.

While action prediction difficulties are a plausible cause of reduced IS in autism, there is no direct evidence of this relation. However, there is evidence that atypical action prediction in autism contributes to reduced co-ordination in joint action, where two people co-ordinate their actions to achieve a shared goal. Autistic and non-autistic children coordinated equally well with an experimenter when predictive demands were minimised because the end point of an action was unambiguous. In contrast, when the experimenter’s movement had to be inferred from kinematic cues alone, autistic children were significantly impaired in their co-ordination (Fulceri et al., 2018). In the context of IS, many movements are likely to be non-transitive and thus lack a clear end point. Less frequent action prediction in autism, and less accurate action prediction, both by autistic people and their non-autistic social partners, may therefore contribute to reduced IS in autism.

### Motor Behaviour

A key component of IS, described in Part 1, is the planning and execution of accurate and timely motor activity. Impairments in motor behaviour frequently co-occur with autism (for reviews see Fournier et al., 2010; Hocking and Caeyenberghs, 2017; Hudry et al., 2020; Zampella et al., 2021), and are therefore a plausible contributor to reduced IS in autistic populations. However, the evidence to support such a contribution is relatively limited. For example, a number of studies have assessed basic motor synchrony, typically by requiring participants to tap a finger in synchrony with a simple, repetitive stimulus. The results indicate that the ability to synchronise simple motor output with basic and non-social stimuli in autism is broadly intact (Koehne et al., 2016; Tryfon et al., 2017; Morimoto et al., 2018; Honisch et al., 2021; Vishne et al., 2021), or even enhanced (Edey et al., 2019).

Further, while there is some evidence of a positive association between motor abilities, assessed in an individual context, and IS (Brezis et al., 2017), the existence of such an association has also been found to depend on the particular tasks used (Fitzpatrick et al., 2017a). Other studies have failed to find a significant association between motor abilities assessed in a solo context and IS in autism (Kaur et al., 2018; Koehler et al., 2021). However, these studies used generalised measures of motor ability, rather than specific component processes of motor functioning (Gowen and Hamilton, 2013), such as motor planning, motor timing, and motor control. The heterogeneity in study outcomes may therefore reflect differential loading of motor tasks onto particular aspects of motor functioning. There is some evidence that motor planning may mediate the association between increased autistic traits and reduced IS (Granner-Shuman et al., 2021), but the role of specific component motor processes in IS in autism is otherwise yet to be explored. In sum, the evidence for an association between motor abilities and reduced IS in autism is mixed. Further research is needed to establish whether specific motor processes might be the key contributors.

An additional consideration in relation to motor behaviour and IS is the motor signatures of interacting partners. As outlined in Part 1, the degree of similarity between partners’ motor signatures affects the extent of IS (Hart et al., 2014; Słowiński et al., 2016). The movement patterns of autistic individuals tend to differ from those produced by non-autistic individuals (Bloch et al., 2019), including across gestures (Anzulewicz et al., 2016), head movements (Martin et al., 2018), and gait (Cho et al., 2022). The relative dissimilarity of individual motor signatures between partners may therefore lead to lower levels of IS in interactions between autistic and non-autistic people (Georgescu et al., 2020).

### Monitoring and Adaptation

The IS-relevant processes described in this conceptual analysis are believed to be embedded in a feedback loop, which includes bidirectional monitoring and error correction (Konvalinka et al., 2010; Marieke Van der Steen and Keller, 2013; Shamay-Tsoory et al., 2019; Feniger-Schaal and Warzager, 2020), and facilitates adaptation of motor behaviour to bring about IS. Effective error monitoring likely depends partly on the extent to which interacting partners visually attend to their own and their partner’s behaviour. Atypical social orienting and attention in autism, as discussed above, may compromise this process. Additionally, because IS depends on the relative timing of partners’ behaviour, effective error monitoring also depends on effective detection of co-occurring behaviours (Shamay-Tsoory et al., 2019). The evidence regarding differences in perceptual sensitivity to the relative timing of stimuli in autism is inconclusive (Casassus et al., 2019; Meilleur et al., 2020), and there has been no exploration of whether autistic and non-autistic people differ in their perceptual sensitivity to IS during interactions. Thus the relevance of relative timing abilities for error monitoring in IS in autism is not yet well understood.

Following error monitoring, internal predictive models and motor plans must be efficiently updated (Shamay-Tsoory et al., 2019). Difficulties for autistic people with action prediction (Montobbio et al., 2022) as well motor planning (Granner-Shuman et al., 2021), both discussed above, are likely to be implicated here. As outlined in Part 1, the speed with which updating occurs is also an important consideration. There is evidence that the updating process may occur more slowly in autistic than in typical populations, resulting in lower levels of synchronous behaviour. For example, autistic participants took significantly longer to adjust their finger tapping when the pace of an isochronous stimulus changed, despite demonstrating comparable levels of synchronisation when the pace of the stimulus was constant (Vishne et al., 2021). This indicates that performance differences were attributable either to slower updating of the internal model, or slower incorporation of the model into updated movement plans (Vishne et al., 2021). Slow updating and error correction within the dynamic context of a social exchange are likely to result in difficulty both in establishing and in maintaining IS, and are therefore plausible contributors to reduced IS in autism.

### Attentional Load

Substantial evidence suggests that attentional resources are atypically distributed in autism. Despite evidence of enhanced perceptual capacity overall (e.g., Remington et al., 2009; Bayliss and Kritikos, 2011), autistic people have been found to display reduced sensitivity to incoming information, narrowly focussed attention to particular stimuli, and difficulties disengaging from stimuli (for reviews see Allen and Courchesnel, 2001, and Keehn et al., 2013). Atypical patterns of attending are likely to lead to a reduction in the resources available for attending to social information relevant to IS, and lead to reduced IS as a result.

As discussed in Part 1, there is evidence that intentional IS is reduced with greater attentional load, through a diminished attentional capacity for IS-relevant stimuli (Temprado and Laurent, 2004). For autistic people, social interactions may involve multiple attention-demanding phenomena, which are not as exacting for non-autistic people. For example, the online process of understanding the mental states of a conversational partner is a relatively intuitive process for non-autistic people, but autistic people may need to engage in effortful compensatory strategies (Livingston et al., 2020). Autistic people may also engage in effortful behaviours aimed at masking autistic social tendencies, which could include suppressing repetitive behaviours, carefully monitoring their eye contact, or using behavioural rules or conversational scripts to guide social behaviour (Livingston et al., 2019b; Cook et al., 2021a,b). Attending to such strategies during conversation may deplete the attentional resources available to autistic people for attending to IS-relevant cues, leading to reduced IS during the interaction.

The influence of environmental distractors on IS may also be different for autistic people. Autistic people experience a range of unusual sensory experiences, including both hyper- and hypo- sensitivity to sensory features of the environment, as well as sensory seeking of preferred sensory experiences (American Psychiatric Association, 2013). These unusual sensory experiences can cause distress or distraction for autistic people (Robertson and Simmons, 2015) which may reduce their capacity for processing and attending to IS. Further, while neurotypical individuals preferentially process social stimuli even when attentional load from non-social stimuli is relatively high (Lavie et al., 2003), the same effect is not observed in autistic people (Remington et al., 2012). This evidence suggests that environmental distractors are more likely to result in reduced IS in autistic than in typical populations.

Further, it has been proposed that the emergence of spontaneous IS precipitates a *reduction* in attentional load, thus enhancing processing capacity for other stimuli (Koban et al., 2019). Given that lower levels of spontaneous IS are generally observed in autism, fewer attentional resources may be available for processing other social cues arising during the course of an interaction.

Overall, therefore, differences in attentional load and the way it is processed may influence IS in autism in a number of ways. Attentional resources for processing IS-relevant stimuli may be depleted by atypical distribution of attentional resources, the socio-cognitive demands of an interaction, the use of compensatory and camouflaging behaviour, or by atypical processing of the sensory environment. Further, autistic people may be relatively susceptible to distractor stimuli, leading to reduced intentional IS in autism as a result. Finally, lower levels of spontaneous IS in autism are likely to place relatively high attentional demands on autistic people, who are likely to experience a reduced capacity for processing other stimuli, including other social information, as a result.

### Social Context

Autism is a condition characterised by atypical social communication, thus the social context is potentially highly relevant to differences in IS in autism. Differences in social orienting and in socially relevant attentional load during interactions, as well as their potential impact on IS, have already been considered above. A broader question concerns whether atypical social communication in autism leads to reduced IS; whether reduced IS may itself give rise to disrupted social communication; or whether there is a complex and bidirectional relationship between the two constructs.

In support of the former proposition, some researchers have proposed that social context is less relevant to autistic people because of a reduced desire to engage in social exchange and to forge social bonds (Chevallier et al., 2012). If social motivation were reduced in autism, this would be likely to have downstream effects on social communication including IS. Just as the desire for social connection is thought to drive IS in typical populations (Miles et al., 2010; Lumsden et al., 2012; Zhao et al., 2015; Brambilla et al., 2016), a reduced desire for social connections could drive reduced levels of IS in autism (Brezis et al., 2017). However, this account of reduced social motivation in autism has been challenged (Jaswal and Akhtar, 2019; Livingston et al., 2019a). There is evidence of substantial variation in levels of social motivation in autism (Garman et al., 2016; Sedgewick et al., 2016), as well as evidence to suggest that levels of social motivation are dissociated from social interaction outcomes (Morrison et al., 2020a). Further, it may be that social motivation is present but atypically expressed in autism (Jaswal and Akhtar, 2019; Livingston et al., 2019a). However, conventional markers of social motivation, such as eye contact (Akhtar and Jaswal, 2020), are themselves likely to facilitate IS (see above). It may be that the absence of such markers, rather than the absence of social motivation *per se*, plays a role in reduced IS for some autistic individuals.

The social context may also influence IS in autism because of elevated rates of social anxiety disorder among autistic people (Spain et al., 2018; Hollocks et al., 2019). People with social anxiety disorder display reduced IS relative to people without the disorder (Hessels et al., 2018). Several features of social anxiety disorder have been suggested as potential explanations for this reduction in IS, including reduced eye contact (Hessels et al., 2018), reduced non-verbal behaviour such as nodding or gesturing, and increased internally focussed attention (Asher et al., 2020). Elevated levels of anxiety may also precipitate increased levels of emotional arousal (O’Haire et al., 2015), which has been associated with faster motor output during an intentional synchronisation task (Monier and Droit-Volet, 2018). Thus, for autistic people, the contextual effect of increased anxiety or arousal during social situations may exacerbate any underlying differences in the component processes that contribute to IS.

It is also important to consider whether reduced IS may itself give rise to disrupted social communication over time. From a developmental perspective, it is possible that early difficulties with the perceptual and motor elements of IS initiate a developmental trajectory whereby early social interactions are experienced as less rewarding by autistic children, such that they are less likely to engage in social interaction. Less engagement would result in fewer opportunities to pick up on social cues and develop typical social skills. In turn, peers and caregivers may find asynchronous interactions with autistic children relatively less rewarding, leading to reduced social engagement from others, further reducing the opportunities for the development of typical social communication abilities (Delafield-Butt et al., 2019; Zampella et al., 2020). Thus, when considering the social context, the relationship between social communication difficulties and reduced IS in autism may in fact be developmental and bidirectional in nature. Longitudinal research is required to understand how these factors influence each other and relate to social communication difficulties over time.

## Discussion

IS is a significant and complex social process which contributes to positive social outcomes and to building social relationships throughout the lifespan. However, its underlying mechanisms and how they relate to one another are still not well understood. In Part 1 of this conceptual analysis, we synthesised a wide range of evidence outlining the contributions of social orienting, multisensory processing, action prediction, and motor planning and execution. We described how these mechanisms are believed to be embedded together in a feedback loop of error monitoring and correction, and reflected on the moderating effects of attentional load and social context. As well as discussing each component mechanism in turn, we also sought to draw out the interdependence between these constructs. A key feature of this interdependence is that one process is likely to have cascading effects on others, such that successful IS depends on each process providing appropriate input for the next. For example, successful action prediction depends in part on an individual’s predictive ability, but cannot take place unless relevant social information has first been gathered *via* efficient social orienting. In Part 2, we applied this understanding of the component processes of IS to consider the factors that might precipitate reduced IS in autism. We outlined evidence of atypical functioning in autism across a number of component mechanisms, and highlighted the variation in the extent to which the evidence supported a link between such divergence in functioning and reduced IS. Overall, however, it is likely that differences across multiple processes contribute to reduced IS in autism, with atypicality in any given process having potential downstream effects on other relevant mechanisms.

Although our analysis described the key component processes that contribute to IS in typical and autistic populations, it is not intended as an exhaustive account of every potential influence on IS. For example, our focus was on non-verbal IS and further consideration should be given to verbal and vocal IS, as well as the complex interplay between non-verbal and verbal IS. Additionally, age-related changes in component processes are likely to influence the extent to which IS occurs. As yet, however, little is known about how developmental timing differences in the emergence of these component skills, and how they vary within autistic and non-autistic populations, influence IS during development.

Similarly, consideration of the neural substrates of IS is likely to shed light on the mechanisms involved in IS. For example, motor cortex activity may play a role in facilitating synchronisation, with evidence of a positive association between levels of sensorimotor activity when observing a partner’s actions and more accurate synchronisation with the partner in finger tapping (Naeem et al., 2012), button pressing (Meyer et al., 2011) and drumming games (Endedijk et al., 2017). Further, increased activation of the motor cortex was observed where participants synchronised with a partner, but not where they synchronised with a non-social stimulus (Novembre et al., 2012), suggesting the motor cortex plays a specific role in facilitating synchronisation with a biological stimulus, rather than synchronisation with external stimuli more generally. Consideration of the neural underpinnings of each component mechanism of IS likely to enrich our understanding as to how IS arises – or is compromised – during social interaction.

Relatedly, synchrony between interacting partners is known to arise at a neural as well as behavioural level (Nam et al., 2020). While there is evidence that neural and behavioural synchrony tend to co-occur and are thought to be closely related (Dumas et al., 2010; Liu et al., 2018; Koban et al., 2019), the mechanisms and directions of influence are not yet fully understood. For example, inducing neural entrainment between partners *via* simultaneous transcranial stimulation of their motor cortices enhanced partners’ finger tapping synchrony (Novembre et al., 2017) and levels of IS in a naturalistic interaction (Pan et al., 2021), suggesting that behavioural synchrony may be preceded and induced by synchrony at a neural level. By contrast, partners who observed themselves acting with IS became neurally synchronised with each other (Levy et al., 2017). Thus, neural entrainment might also arise as a consequence of behavioural entrainment (Wass et al., 2020). Further research is required to understand the potentially bidirectional nature of the relations between these different aspects of synchrony. The relations between neural and behavioural synchrony may also inform our understanding of reduced IS in autism, with some evidence of a dissociation between the two in autistic children (Kruppa et al., 2021).

In the context of autism in particular, the influence of relational factors is also likely to be an important avenue for future research. Our analysis highlights emerging evidence that differences between interacting partners, such as divergent motor signatures and mutual difficulties in action prediction between autistic and non-autistic partners, can impact IS. Further relational considerations are also likely to be relevant. For example, non-autistic people may lack understanding of autistic social behaviour, which may precipitate reduced IS when autistic and non-autistic people interact. Non-autistic people may hold stereotypical assumptions about autistic people and their behaviour, for example, characterising autistic people as unfriendly or odd, or assuming that averted social gaze connotes a lack of social interest (Turnock et al., 2022). Such preconceptions may reduce their inclination to forge a social connection with autistic social partners (Sasson et al., 2017), leading to reduced IS within mixed dyads. Similarly, low levels of acceptance of autistic social behaviour by non-autistic people may impact the extent to which autistic people feel the need to engage in resource-intensive camouflaging behaviours (Livingston et al., 2019b), which may negatively impact on the attentional resources available for processing IS-relevant cues. Few studies to date have examined IS within autistic dyads (Georgescu et al., 2020, being a notable exception). Further investigation of how autistic people synchronise with other autistic people may advance understanding of relational factors and how they might impede IS in mixed interactions.

Future research should also consider the extent to which IS influences social bonding in autism, and its importance to building social relationships relative to other aspects of social behaviour. Existing evidence indicates that autistic people experience equivalent levels of IS (Georgescu et al., 2020) but increased rapport (Crompton et al., 2020a,b; Morrison et al., 2020b) when interacting with other autistic people, relative to when interacting with non-autistic people. Taken together, these findings suggest that, for autistic people, IS may be somewhat dissociated from social bonding. It may be that different aspects of the interaction, such as the extent to which information is efficiently exchanged (Crompton et al., 2020a), or feelings of shared experience (Crompton et al., 2020b), are more important in establishing rapport and bonding for many autistic individuals (Heasman and Gillespie, 2019; Crompton et al., 2020b; Morrison et al., 2020b). Indeed, if different aspects of an interaction are socially salient, then increased allocation of attention to such factors, potentially at the expense of attending to IS-relevant information, might logically be expected. Further investigation is needed to understand the relevance of IS within the broader context of social interaction in autism.

In conclusion, our conceptual analysis has highlighted the importance of understanding both the component processes of IS and the interrelationships between them. Drawing on research that has examined the role of individual component mechanisms, we have provided a framework for understanding how these mechanisms contribute and interact to bring about IS. Our framework has provided a conceptual basis for understanding how non-verbal IS operates in autism and how it relates to autistic experiences of social communication more generally. Finally, there is emerging evidence that IS is reduced in other conditions, including ADHD (Problovski et al., 2021) and schizophrenia (Dean et al., 2021), although the underlying reasons for reduced IS are likely to be different across different disorders. By mapping out the component mechanisms of IS and how they interact, our conceptual analysis may provide a useful starting point for identifying which component mechanisms are uniquely implicated within these different conditions.

## Author Contributions

CB-M, CJ, and EvdH conceived the idea for the article. CB-M, CJ, EvdH, and SG wrote the article. All authors contributed to the article and approved the submitted version.

## Conflict of Interest

The authors declare that the research was conducted in the absence of any commercial or financial relationships that could be construed as a potential conflict of interest.

## Publisher’s Note

All claims expressed in this article are solely those of the authors and do not necessarily represent those of their affiliated organizations, or those of the publisher, the editors and the reviewers. Any product that may be evaluated in this article, or claim that may be made by its manufacturer, is not guaranteed or endorsed by the publisher.
